# NET Release of Long-Term Surviving Neutrophils

**DOI:** 10.3389/fimmu.2022.815412

**Published:** 2022-02-15

**Authors:** Jan Philipp Kolman, Laia Pagerols Raluy, Ingo Müller, Viacheslav O. Nikolaev, Magdalena Trochimiuk, Birgit Appl, Hannah Wadehn, Charlotte Maria Dücker, Fabian David Stoll, Michael Boettcher, Konrad Reinshagen, Julian Trah

**Affiliations:** ^1^Department of Pediatric Surgery, University Medical Center Hamburg-Eppendorf, Hamburg, Germany; ^2^Division of Pediatric Stem Cell Transplantation and Immunology, University Medical Center Hamburg, Hamburg, Germany; ^3^Institute of Experimental Cardiovascular Research, University Medical Center Hamburg-Eppendorf, Hamburg, Germany; ^4^Heinrich Pette Institute, Leibniz Institute for Experimental Virology, Hamburg, Germany

**Keywords:** neutrophil granulocytes, neutrophil extracellular traps, survival, activation, viability

## Abstract

**Background:**

Neutrophil extracellular traps (NETs)—as double-edged swords of innate immunity—are involved in numerous processes such as infection, inflammation and tissue repair. Research on neutrophil granulocytes is limited because of their short lifetime of only a few hours. Several attempts have been made to prolong the half-life of neutrophils using cytokines and bacterial products and have shown promising results. These long-term surviving neutrophils are reported to maintain phagocytic activity and cytokine release; however, little is known regarding their capability to release NETs.

**Methods:**

We analysed the prolongation of neutrophil survival *in vitro* under various culture conditions using granulocyte colony-stimulating factor (G-CSF), lipopolysaccharide (LPS) or tumour necrosis factor alpha (TNF-α) by flow cytometry and a viability assay. Additionally, we assessed NET formation following stimulation with phorbol 12-myristate 13-acetate (PMA) by immunofluorescence staining, myeloperoxidase (MPO)-DNA sandwich-ELISA and fluorometric assays for cell-free DNA (cfDNA), neutrophil elastase (NE) and myeloperoxidase (MPO).

**Results:**

Untreated neutrophils could form NETs after stimulation with PMA for up to 24 h. Incubation with LPS extended their ability to form NETs for up to 48 h. At 48 h, NET release of neutrophils cultured with LPS was significantly higher compared to that of untreated cells; however, no significantly different enzymatic activity of NE and MPO was observed. Similarly, incubation with G-CSF resulted in significantly higher NET release at 48 h compared to untreated cells. Furthermore, NETs showed significantly higher enzymatic activity of NE and MPO after incubation with G-CSF. Lastly, incubation with TNF-α had no influence on NET release compared to untreated cells although survival counts were altered by TNF-α.

**Conclusions:**

G-CSF, LPS or TNF-α each at low concentrations lead to prolonged survival of cultured neutrophils, resulting in considerable differences in NET formation and composition. These results provide new information for the use of neutrophils in long-term experiments for NET formation and provide novel insights for neutrophil behaviour under inflammatory conditions.

## 1 Introduction

Neutrophil granulocytes produce extracellular web-like structures called neutrophil extracellular traps (NETs) which indicate a specialised form of cell death (1). NETs are composed of decondensed chromatin and granule-derived enzymes, such as neutrophil elastase (NE) and myeloperoxidase (MPO) (2). These NETs ensnare pathogens and shield the surrounding tissue from cytotoxic substances while increasing the local concentrations of antimicrobial substances (3). NETs are released up to 4 h after neutrophil stimulation by various inflammatory cytokines and bacterial products, such as interleukin 8 or lipopolysaccharide (LPS) (4). *In vitro*, stimulation with phorbol 12-myristate 13-acetate (PMA) is often used to induce NET formation. PMA leads to NET release *via* direct activation of protein kinase C and is therefore seen as a proof of concept for NET release mechanisms (1, 5). Despite being an anti-pathogen defence mechanism, NETs also have pathological aspects (5–7). Extracellularly, cell-free DNA (cfDNA) and granule-derived enzymes can trigger the production of autoantibodies, thereby promoting autoimmune diseases such as systemic lupus erythematosus (8–10). Furthermore, the web-like structure of cfDNA enhances tumour metastasis (11) and leads to organ damage during sepsis due to its prothrombotic properties (12–15). Wound healing (16), ischaemic reperfusion injuries (17) and ulcerative colitis (18) have also been described to be negatively affected by NETs.

Consequently, several *in vitro* studies examining NET release have been conducted to understand the role of neutrophil granulocytes in inflammation and disease. Neutrophils have a short half-life of 4–9 h and are not yet amenable to long-term analysis through the standard tools of molecular biology, such as transfection (19, 20). The survival of neutrophils was previously prolonged by supplementation with cytokines or bacterial products in low concentrations during cell culture (21–26). One of the frequently used substances, LPS from gram-negative bacteria, is known to activate NET release at high concentrations (3) but was shown to inhibit neutrophil apoptosis at low concentrations (12, 21, 27). A similar dose-dependent behaviour was demonstrated for tumour necrosis factor alpha (TNF-α), with higher concentrations leading to a respiratory burst in neutrophils (22). In addition, granulocyte colony-stimulating factor (G-CSF) promotes neutrophil survival by altering protein expression on a transcriptional level *in vivo* and *in vitro* (28–30). Several studies have demonstrated the preserved functionality of neutrophils cultured with G-CSF, LPS or TNF-α by measuring the production of reactive oxygen species (ROS), ability to interact with endothelial cells or protein biosynthesis (21, 22, 31). As previous studies have described a successful transfection of neutrophils after prolonged survival (32), the question arises whether NET release is preserved over time of survival to conduct NET research with those long-term surviving neutrophils.

Despite all negative aspects of NETs that have been unveiled over the last years, NETs still play an important role in host defence against pathogens, demonstrated by overwhelming infections in patients with chronic granulomatous disease (CGD) where neutrophils are unable to produce NETs (33, 34). Overwhelming bacterial infections in neutropenic or CGD patients are targeted by transfusion of neutrophil granulocytes mobilised by G-CSF and stored for up to 24 h (35–37). Understanding the NET-related behaviour of stored neutrophils could optimise the transfusion outcomes, whereas the benefits of neutrophil transfusion are still discussed (38, 39). Additionally, the modulation of neutrophil survival is reported in sepsis (40), whereas LPS is used in classic sepsis models to induce overwhelming immune response (41). TNF-α, in contrast, plays a major role in ulcerative colitis, whereas NETs sustain inflammatory signals, and neutrophils have also been reported to show increased viability (18, 42).

The current study aimed to investigate the isolated effects of G-CSF, LPS or TNF-α on neutrophil survival, viability and activation and to determine whether the surviving neutrophils can still produce NETs when stimulated by PMA. These insights regarding the behaviour of long-term surviving neutrophils on behalf of NET formation may contribute to further unveiling the role of neutrophils in inflammation and disease.

## 2 Methods

### 2.1 Isolation of Neutrophil Granulocytes

Blood samples were taken after informed, signed consent was obtained from healthy local donors following approval by the Ethics Committee of the Hamburg Medical Association (PV5921). Neutrophil granulocytes were isolated using the MACSxpress^®^ Whole Blood Neutrophil Isolation Kit, human (Miltenyi Biotec, Bergisch Gladbach, Germany) according to the manufacturer’s protocol. Residual erythrocytes were lysed as described before (43). Purity of the extracted neutrophils (>95%) was assured *via* fluorescence-activated cell sorting (FACS) using the anti-CD15-FITC (mAb HI98, IgM) and anti-CD16-PerCP (mAb 3G8, IgG1) antibodies (BioLegend, San Diego, CA, USA). Cell morphology was analysed by haematoxylin and eosin staining.

### 2.2 Culture and Treatment of Neutrophils

After purification, cells were incubated with RPMI medium containing 1% BSA (medium) at 37°C and 5% CO_2_ (untreated control). Treatment was performed by supplementing with G-CSF at 50 U/ml, 500 U/ml or 5000 U/ml (Chugai Pharma, Tokyo, Japan); LPS at 10 ng/ml, 100 ng/ml or 1 µg/ml (Sigma-Aldrich, Saint Louis, MO, USA); or TNF-α at 0.1 ng/ml, 1 ng/ml or 10 ng/ml (Thermo Fischer, Waltham, MA, USA) to the medium mentioned above. After 6 h, 24 h, 48 h and 72 h of incubation, cells were stimulated with 20 nM PMA (Cayman Chemical, Ann Arbor, MI, USA) for 4 h to conduct the NET-related experiments. Cell counts after 6 h, 24 h, 48 h and 72 h of incubation are displayed in **Supplementary Table 1**. Counting was performed with a haemocytometer.

### 2.3 FACS Analysis

We seeded 3 × 10^5^ cells per well in flat-bottom 48-well plates to a final volume of 500 µl, and treated them as mentioned above. After incubation with endpoints at 6 h, 24 h, 48 h and 72 h, cells were washed twice with PBS (Thermo Fischer, Waltham, MA, USA) and labelled with propidium iodide (PI) and Annexin-V-FITC (Becton, Dickinson and Company, Franklin Lakes, NJ, USA) for the detection of necrosis and apoptosis, whereas double negative cells were considered vital. Measuring neutrophil survival by FACS analysis heavily depends on pre-analytical factors such as physical stress by centrifugation. This may lead to false negative measurements (44). To overcome these effects, staining protocols for FACS analysis were altered to reduce the washing steps after staining while adapting staining concentrations to avoid false positives. Furthermore, the neutrophil activation was assessed by staining neutrophils with anti-CD11b-VioBlue (mAb REA713, IgG1) and anti-CD66b-APC (mAb REA306, IgG1) antibodies (Miltenyi Biotec, Bergisch Gladbach, Germany) as described in the manufacturer’s protocols. A positive control treated for 15 min with 20 nM PMA was included (45). Analysis was performed with a flow cytometer (FACSCanto™ II, Becton, Dickinson, and Company, Franklin Lakes, NJ, USA). Data were analysed using BD FACSDiva™ (Becton, Dickinson, and Company, Franklin Lakes, NJ, USA).

### 2.4 Cell Viability Assay

The viability of neutrophils was determined using RealTime-Glo™ MT Cell Viability Assay (Promega, Fitchburg, WI, USA) continuously over 72 h as described in the manufacturer’s protocol. The assay performed in this study is based on the reduction capability of cells, allowing a reduction of a cell-membrane-permeable pro-substrate by the neutrophils. Briefly, 10^5^ cells per well were cultured in a white, clear bottom 96-well plate prior to the addition of the assay test compound to a final volume of 200 µl. Luminescence was measured using a microplate reader (Flex Station^®^ 3, Molecular Devices, San Jose, CA, USA) measuring luminescence with an integration time of 0.5 s at 6 h, 24 h, 48 h and 72 h of incubation. To reduce the variability in the redox capability of BSA (46) caused by oxidation with air, RPMI 1640 medium containing 1% BSA was freshly prepared prior to each experiment.

### 2.5 Assay for Reactive Oxygen Species (ROS)

PMA-induced ROS were measured as described elsewhere (47). Briefly, 5 × 10^4^ cells per well were seeded in black, clear flat-bottom 96-well plates to a final volume of 200 µl and cultured with endpoints as mentioned above. After adding 20 nM PMA and 25 µM Dihydrorhodamine 123 at the respective timepoints, fluorescence was measured after 3 h at 37°C at wavelengths of 505 nm for extinction, 534 nm for emission, and with an automatic cut-off using a microplate reader (Flex Station^®^ 3, Molecular Devices, San Jose, CA, USA).

### 2.6 DNA Immunofluorescence Staining

We seeded 2 × 10^5^ cells per well into 12-well plates containing coverslips to a final volume of 1440 µL and cultured the cells with the endpoints mentioned above. After adding PMA, cells were washed twice with PBS, fixated with 99% methanol, and stored at -20°C. Finally, neutrophils were washed, stained with 1 µg/mL DAPI (Carl Roth GmbH + Co. KG, Karlsruhe, Germany) and mounted with Fluoromont-G (SouthernBiotech, AL, USA). Imaging was performed using the Olympus BX 60 Microscope (Shinjuku, Tokyo, Japan) at 40× magnification. Images were processed with Adobe Photoshop 21.6.2 (San José, CA, USA).

### 2.7 NET Release Assays

#### 2.7.1 Sample Preparation

NET samples were produced according to the instructions of the NETosis Assay Kit (Cayman Chemical, Ann Arbor, MI, USA). Briefly, 3.6 × 10^5^ cells per well were plated in clear 48-well plates and cultured with endpoints as mentioned above followed by two washing steps with NET Assay Buffer (RPMI 1640 containing 1% BSA and 1 mM CaCl_2_). Subsequently, NETs were disrupted by S7 nuclease (Cayman Chemical, Ann Arbor, MI, USA) and collected. Samples were stored at -20°C for up to two weeks.

#### 2.7.2 cfDNA

Amount of cfDNA in the NET samples was measured as described elsewhere (48), replacing SYTOX Green with SYTOX Orange (Thermo Fischer, Waltham, MA, USA). Fluorescence was measured immediately at 544 nm for extinction and 590 nm for emission and with a cut-off at 570 nm using a microplate reader (Flex Station^®^ 3, Molecular Devices, San Jose, CA, USA). The amount of cfDNA was determined relative to a lambda-DNA standard curve. For each treatment, the delta of expelled cfDNA after PMA stimulus and accumulated cfDNA without PMA stimulus is displayed to eliminate the background produced by cell death.

#### 2.7.3 MPO-DNA Sandwich-ELISA

NET-specific MPO-DNA complexes were measured as described elsewhere (49). NET samples and NET standards were diluted 1:50 and 1:20 in PBS with 2.5 mM EGTA (Sigma-Aldrich, Saint Louis, MO, USA), respectively. The anti-MPO capture antibody (ab267425, abcam, Cambridge, UK) was diluted 1:250. Blocking was performed using PBS containing 5% BSA (Sigma-Aldrich, Saint Louis, MO, USA) for 2 h at room temperature. Overnight incubation of samples was performed at 4°C on an orbital shaker at 25 rpm. The Anti-DNA Peroxidase detection antibody (Cell Death Detection ELISA^PLUS^, 11774425001, Sigma-Aldrich, Saint Louis, MO, USA) was diluted 1:500. Then, 15 minutes after addition of tetramethylbenzidine (TMB, Sigma-Aldrich, Saint Louis, MO, USA), the reaction was stopped by adding 2 M H_2_SO_4_. The absorbance was measured at 450 nm with a microplate reader (Flex Station^®^ 3, Molecular Devices, San Jose, CA, USA). The number of MPO-DNA complexes was determined relative to the NET standard curve, which was created by mixing nuclease-digested NET supernatants of PMA-stimulated neutrophils from 5 different donors, as described before (49), and subsequently diluting this mix 1:2 afterwards. NET samples were freshly thawed before every measurement. For each treatment, the delta of expelled MPO-DNA after PMA stimulus and accumulated MPO-DNA without PMA stimulus is displayed to eliminate the background produced by cell death.

#### 2.7.4 Analysis of NE and MPO Activity

Activity of NE in the NET samples was measured according to the instructions “Performing the Elastase Activity Assay” of the NETosis Assay Kit from Cayman Chemical (Ann Arbor, MI, USA). The absorbance was measured after 2 h at 405 nm with a microplate reader (Flex Station^®^ 3, Molecular Devices, San Jose, CA, USA). NE activity was determined relative to a NE standard curve. For each treatment, the delta of NE activity after PMA stimulus and accumulated NE activity without PMA stimulus was displayed to eliminate the background produced by cell death or degranulation.

The activity of MPO in the NET samples was measured according to the instructions “Performing the Assay” of the Neutrophil Myeloperoxidase Activity Assay Kit from Cayman Chemical (Ann Arbor, MI, USA). We purchased 4-aminobenzhydrozide (4-ABH) and tetramethylbenzidine (TMB) from Sigma-Aldrich (Saint Louis, MO, USA). Absorbance was measured after 30 min at 650 nm using a microplate reader (Flex Station^®^ 3, Molecular Devices, San Jose, CA, USA). MPO activity was determined relative to an MPO standard curve. For each treatment, the delta of MPO activity after PMA stimulus and accumulated MPO activity without PMA stimulus is displayed to eliminate the background due to cell death or degranulation.

### 2.8 Statistical Analysis

Statistical analysis was performed with SPSS Statistics 24 (IBM, Armonk, NY, USA) and GraphPad Prism 8 (GraphPad Software, San Diego, CA, USA). Normality of the data was confirmed by the Shapiro-Wilk test with α set at 0.05. Differences between the untreated control group and the treated group each were calculated individually using the student’s t-test. Differences arising from repeated measurements were calculated using ANOVA with Dunnett’s Multiple Comparison. Levels of significance were set at < 0.05 (*), < 0.01 (**), < 0.001 (***). All values represent means ± standard deviation (SD) with n being the number of biological replicates, whereas every measurement was performed as technical duplicates. Means of technical replications were calculated before statistical analysis.

## 3 Results

### 3.1 Modulation of the Survival and Reduction Capability of Neutrophils by G-CSF, LPS, or TNF-α

**Figure 1:**
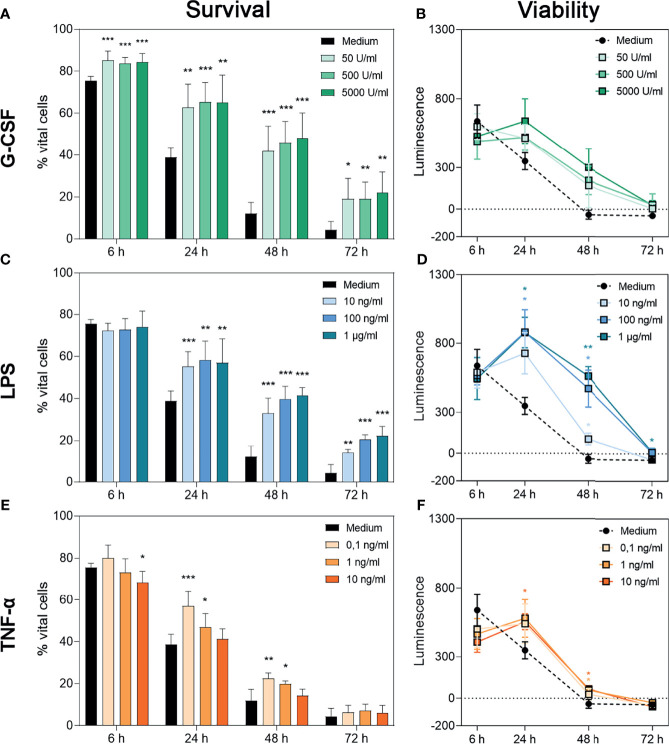
Survival and metabolic activity of neutrophil granulocytes after treatment with G-CSF, LPS or TNF-α. Survival of neutrophils is represented by the percentage of PI- and Annexin V-double negative cells. Neutrophils were incubated with several amounts of G-CSF **(A)**, LPS **(C)**, TNF-α **(E)** or with only medium and analysed after 6 h, 24 h, 48 h and 72 h of incubation by FACS analysis. Values represent means ± SD of n = 6. Differences between the untreated control group and one treated group each were calculated individually using student’s t-test. Levels of significance were set at < 0.05 (*), < 0.01 (**), < 0.001 (***). Regulation of neutrophil viability was measured by bioluminescence. For this purpose, cells were cultured with luciferase and prosubstrate and incubated with G-CSF **(B)**, LPS **(D)**, TNF-α **(F)** or with only the medium for 6 h, 24 h, 48 h and 72 h. Values represent means ± SD of n = 3. Statistical significance for repeated measurements was analysed by ANOVA with Dunnett’s multiple comparison. Levels of significance were set at < 0.05 (*), < 0.01 (**), < 0.001 (***).

To assess the isolated effects of G-CSF, LPS or TNF-α on the survival of neutrophil granulocytes, flow cytometric analysis of non-necrotic (PI^neg^) and non-apoptotic (Annexin-V^neg^) cells was performed (**Figure 1A, C, E**). Representative dot plots for each treatment are displayed in **Supplementary Figures 1**–**3**. Furthermore, the metabolic activity of the surviving cells was evaluated over 72 h through a bioluminescence-based cell viability assay, in which the measured luminescence is proportional to the reduction capability of cells (**Figures 1B, D, F**). In case of neutrophils, the cumulative reduction capability can be altered by the total number of surviving cells, by activation of the surviving cells or by superoxide anion production (50).

After treatment with G-CSF, LPS or TNF-α, neutrophils showed significantly increased survival rates over time compared to cells incubated with the medium alone (**Figures 1A, C, E**). Whereas neutrophils incubated with G-CSF or LPS showed this effect up to 72 h in a dose-independent manner (**Figures 1** **A, C**), cells incubated with TNF-α showed higher survival rates up to 48 h post-treatment with higher doses dampening this effect (**Figure 1E**), as described by van den Berg et al. (22). The survival rates of the untreated neutrophils are compatible with the findings of Monceaux et al. (32). Interestingly, although G-CSF led to a significantly higher neutrophil survival at all timepoints compared to cells treated with medium alone, the cumulative reduction capability of surviving cells was not significantly increased compared to cells treated only with medium (**Figures 1A, B**, respectively).

Concordantly and as depicted in **Figures 1D, F**, analysis of reduction capability demonstrated significantly higher values in cells treated with LPS and TNF-α after 24 h and 48 h but not after 6 h and 72 h. Regarding LPS 24-h treatment with 100 ng/mL and 1 µg/mL resulted in significantly increased bioluminescence levels (**Figure 1D**). At the time point of 48 h, all concentrations led to a significantly higher reduction capability. Even after 72 h incubation with 1 µg/mL LPS, significantly increased reduction capability was observed (**Figure 1D**). After 24 h, neutrophils incubated with 10 ng/mL TNF-α showed a significantly higher reduction capability compared to untreated cells (**Figure 1F**). After 48 h of incubation with 10 ng/mL or 1 ng/mL TNF-α, levels of reduction capability were higher compared to untreated cells (**Figure 1F**).

The discrepancy between higher survival counts and low levels of reduction capability can be explained by the phenomenon of exhausted neutrophils (51). Therefore, we interpreted the whole cell population as exhausted at 48 h for untreated cells. Treatment with G-CSF, LPS or TNF-α postponed this status till 72 h.

### 3.2 Activation of Neutrophil Granulocytes After Treatment With G-CSF, LPS, or TNF-α

**Figure 2:**
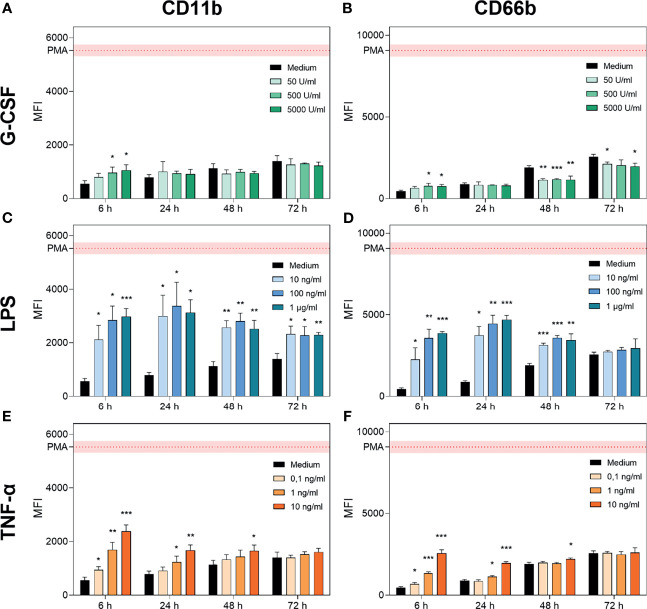
G-CSF, LPS and TNF-α regulate CD11b and CD66b expression in neutrophils. CD11b and CD66b expression levels were analysed by FACS after incubation of neutrophil granulocytes with G-CSF **(A, B)**, LPS **(C, D)**, TNF-α **(E, F)** or with only the medium for 6 h, 24 h, 48 h and 72 h. Activation by a positive control (PMA) was included for reference. Values are represented as means ± SD of, n = 3 of the mean fluorescence intensity (MFI). Differences between the untreated control group and one treated group each were calculated individually by student’s t-test. Levels of significance were set at < 0.05 (*), < 0.01 (**), < 0.001 (***).

To distinguish whether the reduction capability mentioned above was increased by neutrophil survival or activation, the expression of CD11b (adhesion) (52, 53) and CD66b (secondary granules, neutrophil-specific) (54, 55) was analysed using FACS analysis (**Figure 2**). Representative histograms for each treatment are displayed in **Supplementary Figures 1**–**3**. We observed increasing levels of CD11b and CD66b over 72 h of cell culture for cells treated only with medium (**Figure 2**) which match previous findings concerning CD11b (56).

G-CSF supplementation resulted in significantly elevated signs of activation after 6 h for higher concentrations (500 U/mL and 5000 U/mL; **Figures 2A, B**). In contrast, at later timepoints, G-CSF supplementation resulted in maintained activation states with significantly reduced expression of CD66b after 48 h and 72 h of incubation (**Figures 2A, B**). These findings are compatible with the previously described reduced expression of intermediate filaments on neutrophils triggered by G-CSF (57).

In contrast, LPS treatment significantly increased the expression of CD11b and CD66b between 6 h and 48 h, achieving the maximal signal at the 24 h time point. Interestingly, LPS-mediated CD11b increase occurred in a dose-dependent manner after 6 h of incubation, whereas for CD66b, this effect was prolonged up to 24 h post-treatment (**Figures 2C, D**). Additionally, the signal intensity of CD11b but not of CD66b was increased after 72 h of incubation with LPS compared to medium-only treated neutrophils. Activation of neutrophils by LPS was reported to be triggered by even lower doses of 1 ng/mL LPS (12). TNF-α treatment resulted in a dose-dependent increase in CD11b and CD66b expression levels after 6 h (**Figures 2E, F**). CD11b upregulation induced by TNF-α was previously shown by van den Berg et al. (22). After 24 h of incubation, the lowest concentration of 0.1 ng/mL showed no significant increase in CD11b and CD66b expression levels. Furthermore, after 48 h of incubation, only the highest concentration of 10 ng/mL showed a significant increase in CD11b and CD66b expression levels (**Figures 2E, F**). After 72 h of incubation with all concentrations of TNF-α, neutrophil activation levels were equivalent to those in the untreated population (**Figures 2E, F**).

Based on these results, we conclude that neutrophils cultured with G-CSF have a prolonged lifetime, maintain their metabolic activity up to 48 h, and are not additionally activated or primed. Neutrophils cultured with LPS show high parameters of activation as well as a prolonged lifetime and maintain metabolic activities up to 48 h. TNF-α treatment resulted in increased survival up to 48 h, with metabolic activity detectable and additional activation induced in a dose-dependent manner at the early timepoints.

### 3.3 Treatment With G-CSF, LPS or TNF-α Does Not Alter Basal Production of ROS But Alters PMA-Induced ROS Production

**Figure 3:**
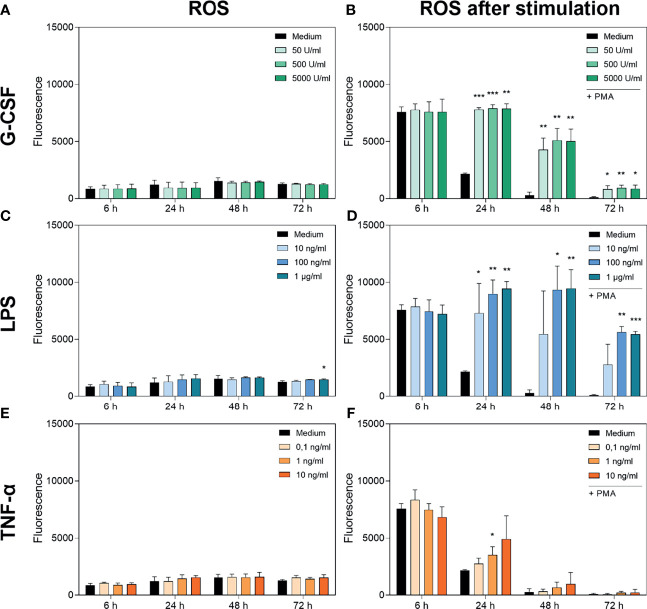
Measurement of ROS accumulation (left panel) and PMA-induced ROS production (right panel) in neutrophils cultured with G-CSF **(A, B)**, LPS **(C, D)** or TNF-α **(E, F)** over 72 h by fluorometric measurement of ROS compared to an untreated control (Medium). Fluorometric measurement was conducted with samples stimulated with 20 nM PMA after 6 h, 24 h, 48 h and 72 h. The displayed fluorescence is proportional to ROS produced. Values represent means ± SD of n = 3. Differences between the untreated control group and one treated group each were calculated individually using student’s t-test. Levels of significance were set at < 0.05 (*), < 0.01 (**), < 0.001 (***).

**Figures 3A, C, E** show the cumulative basal ROS production of neutrophils cultured with G-CSF, LPS or TNF-α over 72 h compared to an untreated group incubated only with medium. Basal ROS production was not significantly altered by any treatment over 72 h. Interestingly, the cumulative basal ROS production remained at a comparable low level over the 72 h of incubation, although the cell numbers were decreasing.

**Figure 3B** shows the PMA-induced ROS production of neutrophils cultured with G-CSF. After 6 h, no significant difference in PMA-induced ROS production was observed. At every later timepoint and every concentration of G-CSF used, the PMA-induced ROS production was significantly higher compared to that in the untreated control. Concordantly, incubation with LPS showed the same dynamic trend except for the lowest concentration of 10 ng/mL LPS (**Figure 3D**). Interestingly, the ROS levels after incubation with LPS are even higher than after incubation with G-CSF. Incubation with TNF-α results in no significantly different ROS production over 72 h compared to the untreated control except after incubation with 1 ng/mL TNF-α for 24 h (**Figure 3F**).

The discrepancy between exhausted cells at 72 h described above and ROS levels still present at this timepoint with G-CSF and LPS enabling PMA-induced ROS production even after 72 h shows that the ROS production of neutrophils does not interfere with the viability assay.

### 3.4 DNA Immunofluorescence Imaging and Quantitative Analysis of cfDNA and MPO-DNA Complexes Suggest a G-CSF- and LPS-Dependent, But Not TNF-α-Dependent, Modulation of NET Formation

To determine whether the surviving neutrophils maintained their ability to produce NETs, we performed immunofluorescence staining of PMA-treated neutrophils incubated with G-CSF, LPS or TNF-α to visualise extracellular web-like DNA, which is the main element of NETs. Additionally, cell-free DNA was quantified through fluorometric analysis under the same culture conditions. A further indication for NET formation was the presence of MPO-DNA complexes detected by a sandwich-ELISA.

**Figure 4:**
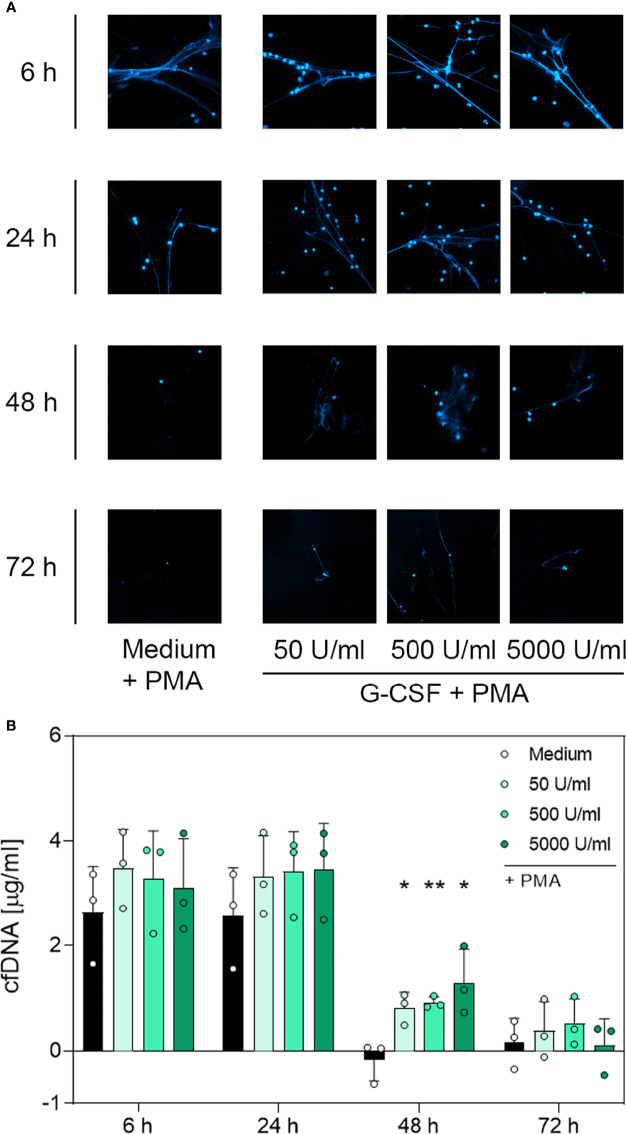
Immunofluorescence imaging of extracellular DNA and quantitative analysis of cfDNA induced by PMA after incubation with G-CSF. **(A)** Immunofluorescence imaging shows extracellular DNA of neutrophils cultured with 50 U/mL, 500 U/mL, 5000 U/mL of G-CSF or of an untreated control (medium). Cells were stimulated with 20 nM PMA at the indicated time points. After fixation, DNA was stained with 1 µg/mL DAPI (blue). PMA-induced NET formations can be detected after up to 48 h after treatment with G-CSF. Images represent areas with comparable cell density at 40× magnification. **(B)** Quantification of PMA-induced cfDNA release by fluorometric measurement of cfDNA after treatment of neutrophil granulocytes with 50 U/mL, 500 U/mL or 5000 U/mL of G-CSF compared to untreated control (medium). Fluorometric measurement was performed with samples stimulated with 20 nM PMA after 6 h, 24 h, 48 h and 72 h. G-CSF preserves cfDNA release for up to 48 h with significant difference compared to the untreated group. Values represent means ± SD of n = 3. Differences between the untreated control group and one treated group each were calculated individually using student’s t-test. Levels of significance were set at < 0.05 (*), < 0.01 (**).

**Figure 5:**
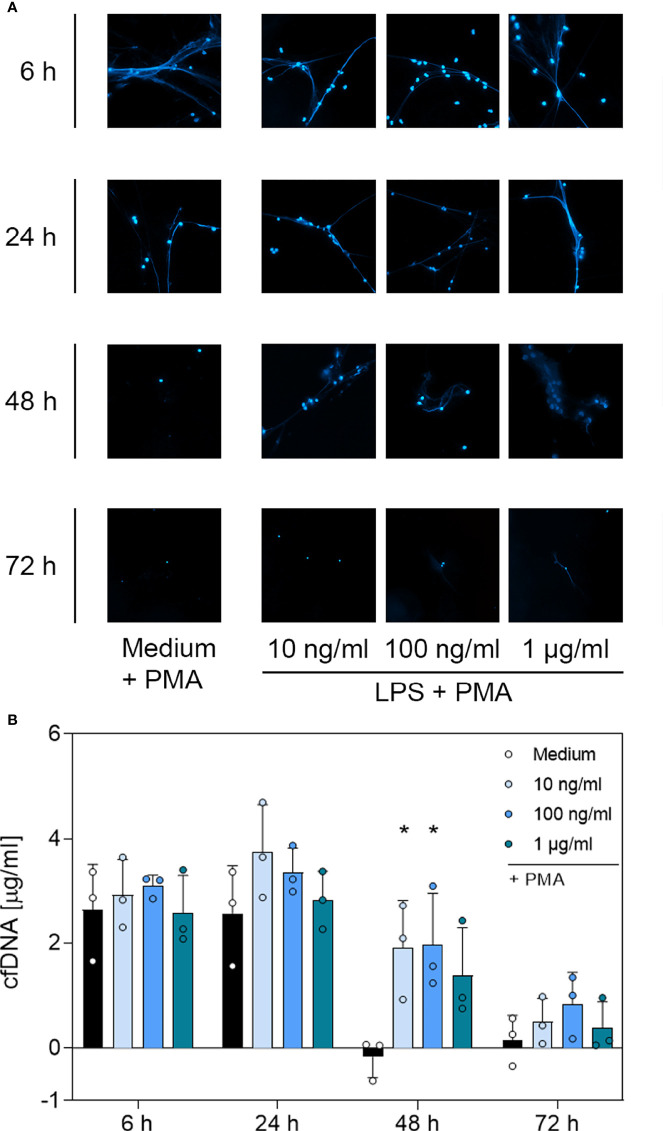
Immunofluorescence imaging of extracellular DNA and quantitative analysis of PMA-induced cfDNA after incubation with LPS. **(A)** Immunofluorescence imaging shows extracellular DNA of neutrophils cultured with 10 ng/mL, 100 ng/mL, 1 µg/mL of LPS or of an untreated control (medium). Cells were stimulated with 20 nM PMA at the indicated time points. After fixation, DNA was stained with 1 µg/mL DAPI (blue). PMA-induced NET formations can be detected after up to 48 h after treatment with LPS. Images represent areas with comparable cell density 40× magnification. **(B)** Quantification of PMA-induced cfDNA release by fluorometric measurement of cfDNA after treatment of neutrophil granulocytes with 10 ng/mL, 100 ng/mL or 1 µg/mL LPS compared to an untreated control (medium). Fluorometric measurement was conducted with samples stimulated with 20 nM PMA after 6 h, 24 h, 48 h and 72 h. LPS preserves cfDNA release for up to 48 h with significant differences compared to the untreated group at 48 h. Values represent means ± SD of n = 3. Differences between the untreated control group and one treated group each were calculated individually by student’s t-test. Levels of significance were set at < 0.05 (*).

**Figure 6:**
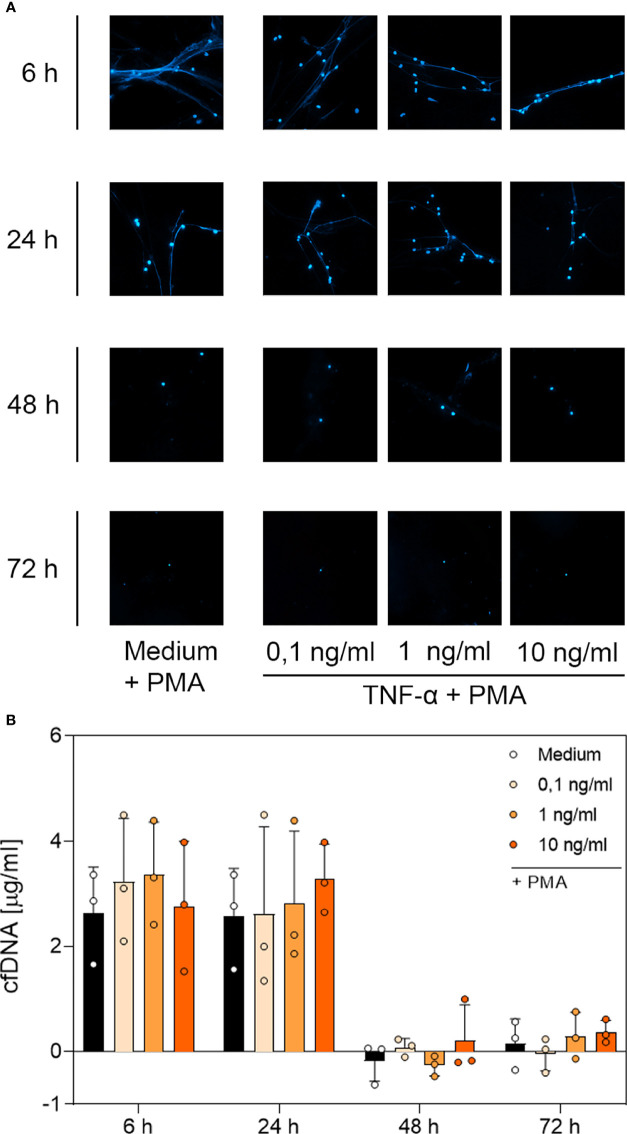
Immunofluorescence imaging of NETs and quantitative analysis of PMA-induced cfDNA after incubation with TNF-α. **(A)** Immunofluorescence imaging shows extracellular DNA of neutrophils cultured with 0.1 ng/mL, 1 ng/mL, 10 ng/mL of TNF-α or of an untreated control (medium). Cells were stimulated with 20 nM PMA at the indicated time points. After fixation, DNA was stained with 1 µg/mL DAPI (blue). PMA-induced NET formations can be detected after up to 24 h after treatment with TNF-α. Images represent areas with comparable cell density at 40× magnification. **(B)** Quantification of PMA-induced cfDNA release by fluorometric measurement of cfDNA after culturing neutrophil granulocytes with 0.1 ng/mL, 1 ng/mL or 10 ng/mL TNF-α compared to an untreated control (medium). Fluorometric measurement was conducted with samples stimulated with 20 nM PMA after 6 h, 24 h, 48 h and 72 h. TNF-α preserves cfDNA release for up to 24 h with no significant difference compared to the untreated group. Values represent means ± SD of n = 3. Differences between the untreated control group and one treated group each were calculated individually using student’s t-test. Levels of significance set at <0.05 were not reached.

Contrary to unstimulated cultured neutrophils (**Supplementary Figure 4**), immunostaining of PMA-stimulated cells (**Figures 4A**, **5A** and **6A**) showed extracellular DNA strains. Cells incubated with medium only showed PMA-induced extracellular DNA formations after incubation for 6 h and 24 h. Additional application of G-CSF or LPS for culturing prolonged the formation of DNA web-like structures by PMA up to 48 h post-incubation (**Figures 4A** and **5A**, respectively). In contrast, neutrophils treated with TNF-α showed DNA secretion by PMA only up to 24 h (**Figure 6A**). Correspondingly, quantification of cfDNA revealed a significant increase of extracellular DNA after 48 h incubation for all G-CSF concentrations, as depicted in **Figure 4B** and for 10 ng/mL and 100 ng/mL but not 1 µg/mL LPS (see **Figure 5B**). Incubation with TNF-α presented no modification on cfDNA amounts compared to the medium control (**Figure 6B**), whereas after 6 h and 24 h, incubation led to similar levels of cfDNA; little to no cfDNA was detected 48 h and 72 h post-treatment (**Figure 6B**).

Based on the microscopic analysis of PMA-induced NET release combined with the quantitative analysis of cfDNA, we conclude that treatment with G-CSF or LPS preserved NET formation for up to 48 h. Even after 72 h, cells treated with G-CSF or LPS showed single strands of extracellular DNA. Treatment with TNF-α and no treatment resulted in preserved PMA-induced secretion of NET formations for up to 24 h. After 48 h and 72 h, the untreated and TNF-α-treated cells showed no signs of extracellular DNA after stimulation with PMA.

**Figure 7:**
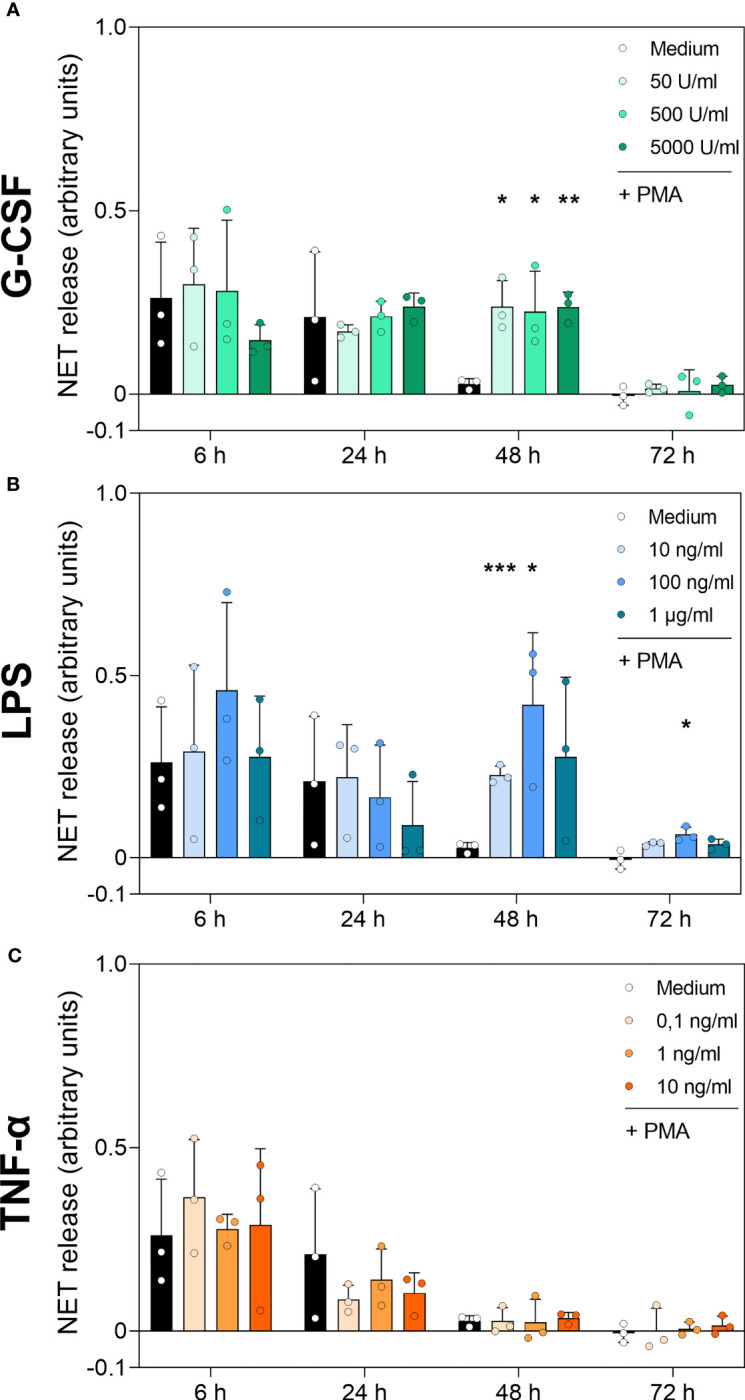
DNA-MPO complexes after stimulation with PMA in neutrophils incubated with G-CSF, LPS or TNF-α. Neutrophils were treated with G-CSF **(A)**, LPS **(B)**, TNF-α **(C)** or medium only for 6 h, 24 h, 48 h and 72 h. Extracellular MPO-DNA complexes were detected by sandwich ELISA: capture antibody was MPO-directed and detection of DNA by antibody for double-stranded DNA coupled with a peroxidase. Results were measured by fluorometry at 650 nm. Values represent means ± SD of n = 3. Differences between the untreated control group and one treated group each were calculated individually using student’s t-test. Levels of significance were set at < 0.05 (*), < 0.01 (**), < 0.001 (***).

Detection of DNA-MPO complexes resulted in significantly increased values for all G-CSF concentrations at 48 h compared to untreated cells (Medium) (**Figure 7A**). LPS treatment resulted in a higher number of DNA-MPO complexes after 48 h of incubation at 10 ng/mL and 100 ng/mL compared to cells cultured with medium alone (**Figure 7B**). Even after 72 h incubation, 100 ng/mL LPS resulted in a significantly increased level of DNA-MPO complexes compared to untreated cells. Finally, neutrophils incubated with TNF-α showed no significant difference in NET release compared to the untreated cell population among all time points (**Figure 7C**). The ELISA results presented above reinforce the data obtained by the immunofluorescence assay and cfDNA assay. Absolute number of DNA-MPO complexes may be underestimated by detachment of MPO from DNA during S7 nuclease treatment.

### 3.5 Qualitative Analysis of NETs by Examining Enzymatic Activity of NET Components

Subsequently, we aimed to elucidate whether the maintained release of NET structures and NET-related complexes was paired with maintained enzymatic activity. For this purpose, the activities of NE and MPO were analysed.

**Figure 8:**
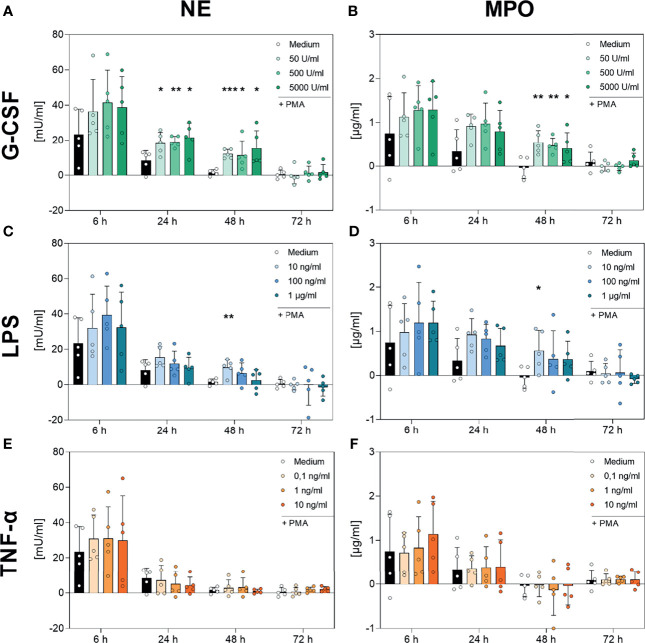
Analysis of NE- and MPO-activity in PMA-induced NETs after incubation with G-CSF, LPS or TNF-α. Neutrophils were incubated with G-CSF **(A, B)**, LPS **(C, D)**, TNF-α **(E, F)** or medium only and stimulated with 20 nM PMA at the indicated timepoints. Neutrophil elastase (left panel) and myeloperoxidase (right panel) activities were measured using the absorption with a plate-reader (405 nm for NE, 650 nm for MPO) and normalized to a standard. Values represent means ± SD of n = 5. Differences between the untreated control group and one treated group each were calculated individually using the student’s t-test. Levels of significance were set at < 0.05 (*), < 0.01 (**), < 0.001 (***).

As shown in **Figure 8**, neutrophils incubated with medium alone showed a decreasing PMA-induced NE and MPO activity over the time, reaching low or not-detectable signals at 48 h and 72 h. NE and MPO activity decreased with decreasing number of surviving cells (**Figures 1** and **8**). Although, the addition of TNF-α to the cell culture showed no modulation on the kinetics of NE (**Figure 8E**, respectively), G-CSF led to significantly increased NE activity at the 24 h and 48 h timepoints for every concentration used (**Figure 8A**). Interestingly, the lowest concentration of LPS (10 ng/mL) also resulted in significantly higher NE activity after 48 h compared to the untreated cells (**Figure 8C**).

Furthermore, **Figure 8** **F** demonstrates no regulation by TNF-α on the activity and kinetics of MPO compared to cells incubated with only medium. Incubation with the lowest used concentration of LPS (10 ng/mL) resulted in a significantly higher activity of MPO after 48 h (**Figure 8** **D**). Strikingly, G-CSF incubation resulted in significantly higher MPO activity after 48 h of incubation at every concentration used (**Figure 8B**).

## 4 Discussion

In the current study, we aimed to evaluate whether long-term surviving neutrophils, cultured with G-CSF, LPS or TNF-α, maintain the ability to form NETs. It is well known that G-CSF prolongs the survival of neutrophil granulocytes *in vitro* (21). In this study, we demonstrated that these surviving neutrophils also maintain reduction capability up to 48 h with no additional elevation of activation markers and can form NETs with sustained enzymatic activity of NE and MPO.

G-CSF is linked to hypoxia-inducible factor 1α (HIF-1α) (58), which works as a transcription regulator of NF-κB and is also a known antiapoptotic stimulus (32, 59). Cell survival and pro-inflammatory activation in neutrophils is regulated by NF-κB. This transcription factor is central to neutrophil function and shows a unique expression pattern distinct from that of other leukocyte subsets (60). HIF-1α was reported to increase CD11b expression in B-cells, which act as suppressors in inflammatory bowel disease (61). As we could show that G-CSF leads to no alterations in the expression of CD11b compared to untreated controls and CD11b is needed and upregulated for neutrophil apoptosis (56, 62), the role of surviving neutrophils should be further investigated in terms of G-CSF, HIF-1α and CD11b.

Another interesting viewpoint might be the new pathway of mitochondrial NETosis (63). Despite the pathway described above, G-CSF was shown to act by blocking the redistribution of the Bcl-2 proteins Bid/Bax and inhibiting caspase activation. Bid/Bax is activated after cell-death activation to induce mitochondrial release of proapoptotic factors (64). Stabilization of this mitochondrial activation pathway might play a role in the preservation of the ability to produce previously described mitochondrial NETosis (63).

Our findings show no significant difference in enzymatic activity of both enzymes (NE and MPO) after G-CSF treatment for any time point other than 48 h compared to untreated cells, although higher counts of surviving cells were achieved at all time points. PMA-induced NET release depends on ROS formed by the NADPH oxidase complex, activated by protein kinase C (1, 2, 65). *In vivo*, protein kinase C is activated by elevated cytosolic Ca^2+^ levels (6). Furthermore, NET release is regulated by the migration of NE and MPO into the nucleus (2). ROS production was demonstrated to be possible after G-CSF-induced neutrophil survival, and the functionality of NADPH oxidase was shown to be preserved after treatment with G-CSF (21, 36). We reproduced these findings. Our study showed intact NE and MPO enzyme activity after G-CSF-induced neutrophil survival. For the cells treated with G-CSF at the earlier time points, the increase in survival and relative decrease in release of NET components may be explained by interference of G-CSF in Ca^2+^ signalling, as G-CSF lowers intracellular Ca^2+^ levels to inhibit apoptosis (66).

We describe preserved NET formation over 48 h with maintained enzymatic activity after G-CSF treatment for the first time. This could have important implications for the clinical use of neutrophil transfusions for neutropenic patients. Considering that higher doses of neutrophils resulted in a better secondary outcome (38), and that our study showed preserved NET release for all concentrations of G-CSF used after 48 h of incubation while considering the necessary ability of neutrophils to produce functional NETs to act successfully *in vivo*, our study provides data to reason clinical studies using G-CSF not only to mobilise neutrophils but also to enhance transport and storage conditions, as already suggested (67). The preserved NET-release ability may improve the clinical outcomes of neutrophil transfusions.

LPS in low doses is also a well-known antiapoptotic stimulus for neutrophils (21). Medium supplementation with LPS, however, results in highly activated neutrophils. These surviving neutrophils also showed NET formations after stimulation with PMA up to 48 h of incubation; however, the enzymatic activity of NE and MPO was compromised at this timepoint. This effect on survival and activation is most likely triggered by the activation of toll-like receptor 4 (TLR4) (68). Interestingly, significantly higher survival of cells after 24 h of cultivation with LPS (**Figure 1C**) did not enable neutrophils to form significantly more MPO-DNA complexes (**Figure 7B**), interpreted as NETs, compared to untreated cells. This might be explained by the exhaustion of cells as described previously. The described exhaustion was coupled to high values of CD11b, concordantly with the results of our study (69).

Interestingly, after 48 h, cells could expel significantly more cfDNA and DNA-MPO complexes in accordance with higher survival rates. These LPS-treated cells had high viability and high activation markers. However, despite the finding of significantly more NET formations, the enzymatic activity was only significantly increased by treatment with the lowest concentration of 10 ng/mL LPS (**Figures 8C, D**). Despite studies suggesting that the binding of NE to DNA inhibits the proteolytic activity of the protease (70), other studies have shown that NET-associated NE remains proteolytically active (71). Considering that LPS-induced neutrophil survival is independent of protein biosynthesis (72), low enzymatic activity in NETs could be explained by degradation over time with no new enzymes synthesized.

Another reason for the high DNA-MPO levels without enzymatic activity after 48 h for the higher concentrations of LPS used could be explained by the high ROS levels triggered in the surviving neutrophils treated with high doses of LPS, as shown in **Figure 3D**. LPS is known to prime neutrophils and leads to ROS production *via* NADPH oxidase (73). High levels of ROS could additionally degrade enzymes and prime the neutrophils. Therefore, the stimulation with PMA resulted in higher levels of DNA-MPO complexes without enzymatic activity.

Interestingly, we could show that the basal ROS production after any treatment used does not differ from the basal ROS production of untreated cells, although PMA-induced ROS production is altered by the treatment. We showed that higher levels of LPS lead to high levels of ROS even after 72 h (**Figure 3D**). This overshooting ROS production after priming with LPS could lead to additional tissue damage (74).

Our findings indicate that the formation of large amounts of NET with loss of enzymatic activity after survival by LPS might furthermore play a role in the process of bacterial sepsis. This could explain how the negative aspects of NETs - namely, leading to septic complications - overtake during systemic inflammation. This should be investigated in further studies, as this could lead to new therapy options for sepsis (13, 40, 75) and the absence of NETs does not increase host vulnerability (76). In contrast, the long-term surviving neutrophils are a relevant factor regarding tissue damage. *In vivo* studies have demonstrated increased tissue damage to be caused by longer neutrophil survival (77). This means that cfDNA accumulation in septic tissue by surviving neutrophils contributes to negative outcomes in sepsis and disease and should be addressed in further studies.

Additionally, neutrophil transfusion with preserved NET release by G-CSF should be discussed as a tentative treatment option for septic patients. Several studies concerning G-CSF treatment of septic patients show better bacterial clearance and improved outcome by restoring neutrophil functions (78). Hemofiltration of cytokines and endotoxins and selective removal of neutrophils show also promising results in treatment of sepsis (79, 80). Future studies should investigate if these effects could benefit from an additional transfusion of functioning neutrophils stored with G-CSF.

Using TNF-α to prolong neutrophil lifetime results in no significantly different behaviour in terms of PMA-induced NET release compared to untreated cells, despite an alteration in the survival count and activation occurring at the earlier timepoints. Neutrophil granulocytes treated with TNF-α showed high survival and viability for up to 48 h (**Figures 1E, F**). High activation markers, especially at the 48 h timepoint, could only be observed for high treatment doses (**Figures 2E, F**). The effect of TNF-α on neutrophils is well characterised and has different effects on the apoptosis of neutrophils depending on the dose (22). An early proapoptotic effect is exerted by tumour necrosis factor receptor 1 (TNFR1) and TNFR2, and a latter survival effect is mediated by the activation of phosphoinositide 3-kinase and NF-κB (81). In addition to this effect, another group hypothesised that TNF-α induces cell death in susceptible cells early after the start of treatment but induces an antiapoptotic pathway in the surviving cells (29). TNF-α at higher doses initiates NET formation, which sustains the inflammatory signals in ulcerative colitis, as shown in neutrophils of patients (18). Interestingly, despite the higher survival up to 48 h by low doses of TNF-α, there is no difference to the untreated cells after 6 h or 24 h of incubation concerning PMA-induced NET formation and no significant difference in the measured NET markers. This could suggest a protective mechanism, in which the survival of neutrophils by low doses of pro-inflammatory cytokines such as TNF-α does not lead to overshooting NET release but still benefits from the longevity of neutrophils at inflammatory sites by use of other neutrophil functions (82).

To date, NET release by primary neutrophils after prolonged cultivation with various cytokines and bacterial products has not previously been reported. Numerous studies have assessed the primary functions of neutrophils, such as ROS production, ability to interact with endothelial cells or protein biosynthesis after prolongation of their lifetime and have described these functions as preserved over the time of survival (21, 22, 31, 32, 72). Observations of neutrophils *in vitro* may not represent the mechanisms *in vivo* because surrounding tissues as well as cell–cell interactions are crucial for neutrophil function *in vivo* (12, 20, 83, 84). Nonetheless, it is not possible to reproduce the complex interactions of neutrophils *in vivo* in a cell culture system. Our study shows that untreated neutrophils can form NETs for up to 24 h of incubation. G-CSF and LPS prolong the ability to form NETs for up to 48 h, whereas LPS treatment results in NETs with low enzymatic activity. Incubation with TNF-α did not result in significantly different NET release compared to the untreated group. We provided the first insight regarding NET release after prolonged survival in our study and developed a possible cultivation method to broaden the amenable methods for the study of neutrophils. Further *in vivo* studies on NET release in long-term surviving neutrophils are required to fully understand their role in disease and therapy.

## Data Availability Statement

The raw data supporting the conclusions of this article will be made available by the authors, without undue reservation.

## Ethics Statement

The studies involving human participants were reviewed and approved by Ethics Committee of the Hamburg Medical Association. The patients/participants provided their written informed consent to participate in this study.

## Author Contributions

JPK and JT conceptualized the study. JPK conducted the investigation. JT, MT, BA, LPR, HW, CMD, and VON curated the execution of the experiments. JPK, FDS, JT, and IM conducted the data curation and performed the formal analysis. JPK and JT wrote the manuscript. LPR, KR, MB, IM, and FDS reviewed, edited, and revised the manuscript. All authors contributed to the article and approved the submitted version.

## Funding

Logistical and financial resources were provided by the Department of Pediatric Surgery, University Medical Center Hamburg-Eppendorf, Martinistrasse 52, 20246, Hamburg, Germany. FACS analysis was performed at FACS Sorting Core Unit, University Medical Center Hamburg-Eppendorf, Martinistrasse 52, 20246, Hamburg, Germany. VON was supported by the Deutsche Forschungsgemeinschaft (SFB 1328).

## Conflict of Interest

The authors declare that the research was conducted in the absence of any commercial or financial relationships that could be construed as a potential conflict of interest.

## Publisher’s Note

All claims expressed in this article are solely those of the authors and do not necessarily represent those of their affiliated organizations, or those of the publisher, the editors and the reviewers. Any product that may be evaluated in this article, or claim that may be made by its manufacturer, is not guaranteed or endorsed by the publisher.

## Supplementary Material

Supplementary Figure 1 Representative FACS dot plots for survival and histograms for activation after treatment with G-CSF. Neutrophils were incubated with several amounts of G-CSF or medium only and analysed after 6 h **(A)**, 24 h **(B)**, 48 h **(C)** and 72 h **(D)** incubation by staining with Annexin-V and PI. Double negative cells (green) are considered vital, Annexin-V^pos^ (orange) are considered in apoptosis and double positive cells (red) are considered dead. Expression of CD11b and CD66b was analysed by FACS analysis after incubation of neutrophil granulocytes with G-CSF or medium only for 6 h **(A)**, 24 h **(B)**, 48 h **(C)** and 72 h **(D)**. Every dot plot or histogram consists of 10.000 events. Gating cut-off was set by measurement of unstained controls for PI and Annexin-V or iso controls for CD11b and CD66b.

Supplementary Figure 2 Representative FACS dot plots for survival and histograms for activation after LPS treatment. Neutrophils were incubated with several amounts of LPS or medium only and analysed after 6 h **(A)**, 24 h **(B)**, 48 h **(C)** and 72 h **(D)** incubation by staining with Annexin-V and PI. Double negative cells (green) are considered vital, Annexin-V^pos^ (orange) are considered in apoptosis and double positive cells (red) are considered dead. Expression of CD11b and CD66b was analysed by FACS analysis after incubation of neutrophil granulocytes with LPS or medium only for 6 h **(A)**, 24 h **(B)**, 48 h **(C)** and 72 h **(D)**. Every dot plot or histogram consists of 10.000 events. Gating cut-off was set by measurement of unstained controls for PI and Annexin-V or iso controls for CD11b and CD66b.

Supplementary Figure 3 Representative FACS dot plots for survival and histograms for activation after TNF-α treatment. Neutrophils were incubated with several amounts of TNF-α or medium only and analysed after 6 h **(A)**, 24 h **(B)**, 48 h **(C)** and 72 h **(D)** incubation by staining with Annexin-V and PI. Double negative cells (green) are considered vital, Annexin-V^pos^ (orange) are considered in apoptosis and double positive cells (red) are considered dead. Expression of CD11b and CD66b was analysed by FACS analysis after incubation of neutrophil granulocytes with TNF-α or medium only for 6 h **(A)**, 24 h **(B)**, 48 h **(C)** and 72 h **(D)**. Every dot plot or histogram consists of 10.000 events. Gating cut-off was set by measurement of unstained controls for PI and Annexin-V or iso controls for CD11b and CD66b.

Supplementary Figure 4 Immunofluorescence imaging of neutrophils. Immunofluorescence imaging showed no extracellular DNA of neutrophils incubated with G-CSF, LPS or TNF-α. After fixation, DNA was stained with 1 µg/mL DAPI (blue). Images represent areas with comparable cell density at 40× magnification.

Supplementary Table 1 Cell count after incubation time is displayed in relation to 3,5 × 10^5^ cells seeded at the beginning. Neutrophils were incubated with mentioned doses of G-CSF, LPS, TNF-α or Medium only and counted after 6 h, 24 h, 48 h and 72 h of incubation with a haemocytometer. Values represent means ± SD of n = 3.

## Abbreviations

NETs, Neutrophil extracellular traps; NE, Neutrophil elastase; MPO, Myeloperoxidase; PMA, Phorbol 12-myristate 13-acetate; cfDNA, cell-free-DNA; LPS, Lipopolysaccharide; G-CSF, Granulocyte colony-stimulating factor; TNF-α, Tumour necrosis factor alpha; ROS, Reactive oxygen species; PBS, Phosphate-buffered saline; BSA, Bovine serum albumin; FACS, Fluorescence-activated cell sorting; PI, Propidium iodide; 4-ABH, 4-Aminobenzhydrozide; TMB, Tetramethylbenzidine; SD, Standard deviation; CGD, Chronic granulomatous disease; TLR4, Toll-like receptor 4; NF-κB, Nuclear factor-κB; TNFR, Tumour necrosis factor receptor; ROS, Reactive oxygen species.
