# Supplementary figures and images for: NET Release of Long-Term Surviving Neutrophils
Source: Front Immunol. 2022 Feb 15;13:815412. doi: 10.3389/fimmu.2022.815412 (PMC8887621; doi:10.3389/fimmu.2022.815412)

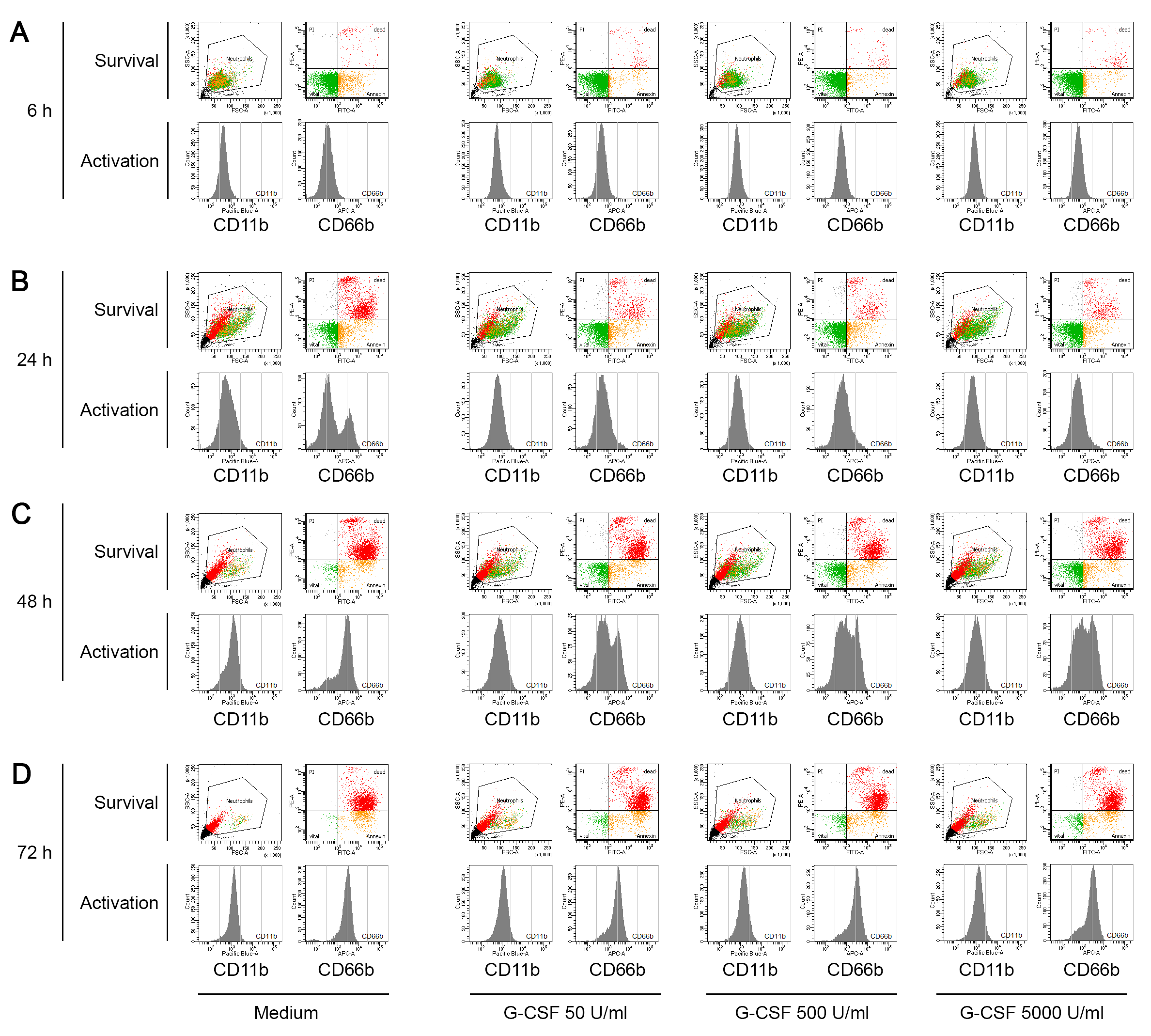

Supplement: Supplementary Figure 1 — Representative FACS dot plots for survival and histograms for activation after treatment with G-CSF. Neutrophils were incubated with several amounts of G-CSF or medium only and analysed after 6 h (A), 24 h (B), 48 h (C) and 72 h (D) incubation by staining with Annexin-V and PI. Double negative cells (green) are considered vital, Annexin-Vpos (orange) are considered in apoptosis and double positive cells (red) are considered dead. Expression of CD11b and CD66b was analysed by FACS analysis after incubation of neutrophil granulocytes with G-CSF or medium only for 6 h (A), 24 h (B), 48 h (C) and 72 h (D). Every dot plot or histogram consists of 10.000 events. Gating cut-off was set by measurement of unstained controls for PI and Annexin-V or iso controls for CD11b and CD66b. [file Image_1.tif]

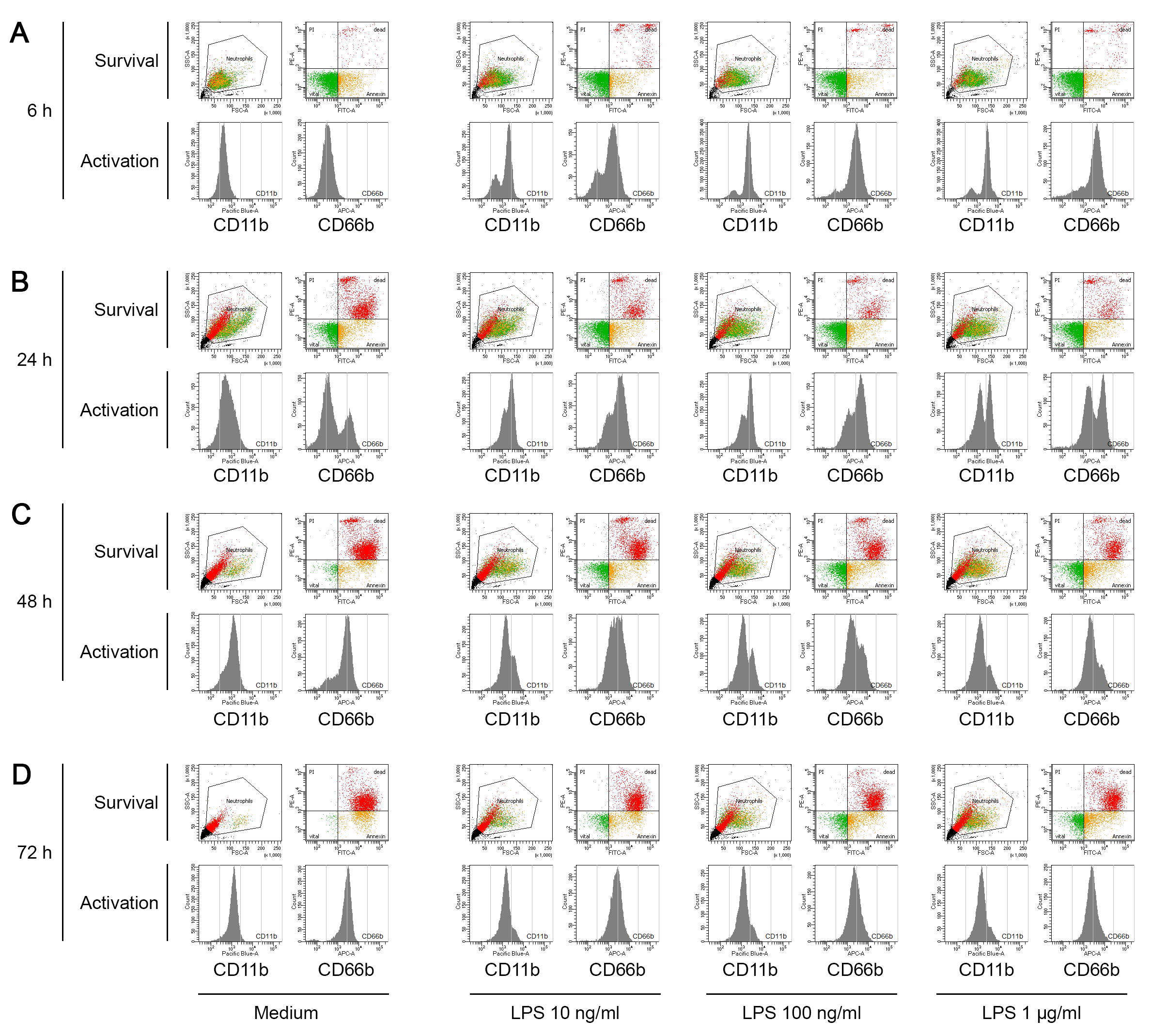

Supplement: Supplementary Figure 2 — Representative FACS dot plots for survival and histograms for activation after LPS treatment. Neutrophils were incubated with several amounts of LPS or medium only and analysed after 6 h (A), 24 h (B), 48 h (C) and 72 h (D) incubation by staining with Annexin-V and PI. Double negative cells (green) are considered vital, Annexin-Vpos (orange) are considered in apoptosis and double positive cells (red) are considered dead. Expression of CD11b and CD66b was analysed by FACS analysis after incubation of neutrophil granulocytes with LPS or medium only for 6 h (A), 24 h (B), 48 h (C) and 72 h (D). Every dot plot or histogram consists of 10.000 events. Gating cut-off was set by measurement of unstained controls for PI and Annexin-V or iso controls for CD11b and CD66b. [file Image_2.tif]

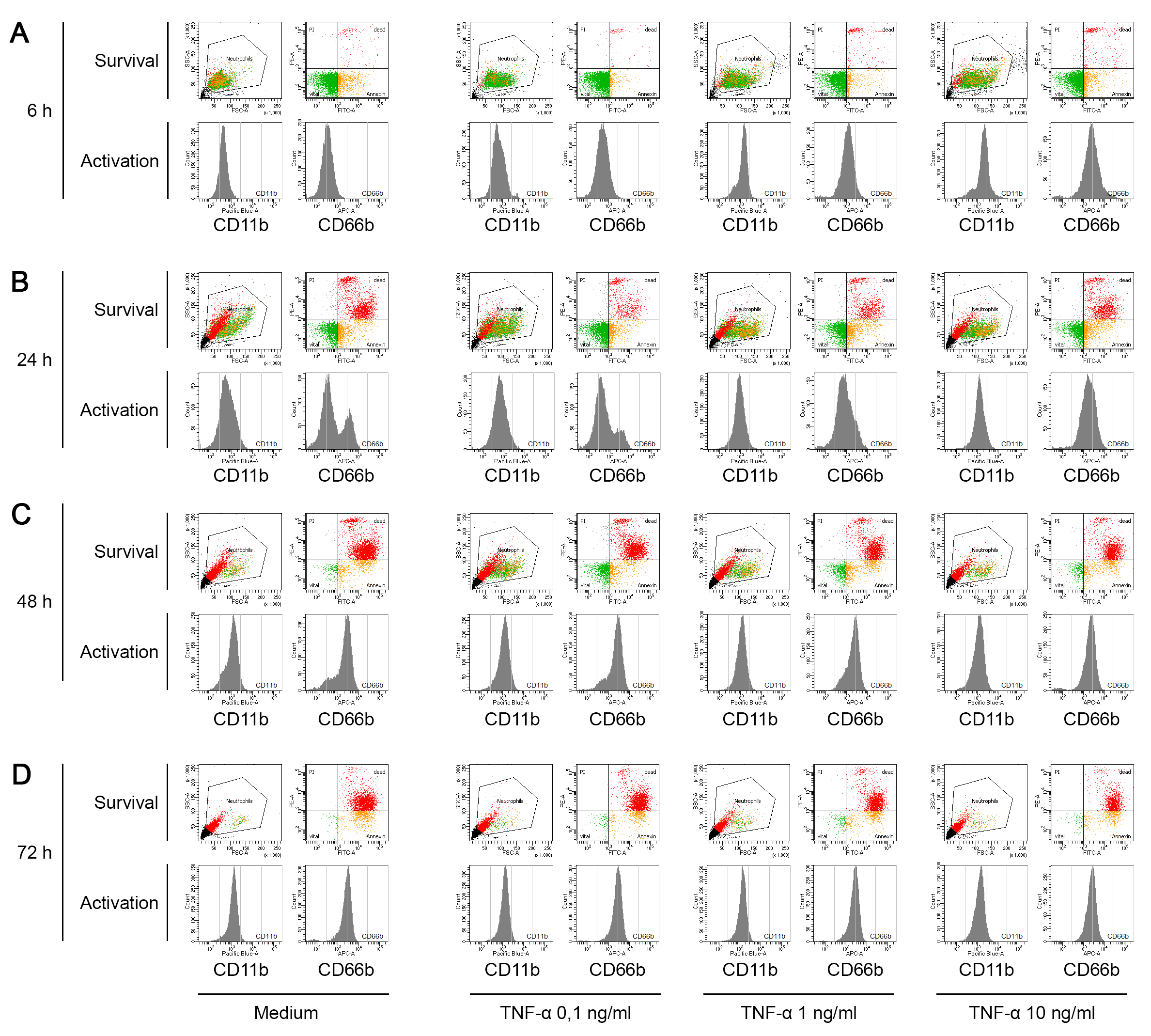

Supplement: Supplementary Figure 3 — Representative FACS dot plots for survival and histograms for activation after TNF-α treatment. Neutrophils were incubated with several amounts of TNF-α or medium only and analysed after 6 h (A), 24 h (B), 48 h (C) and 72 h (D) incubation by staining with Annexin-V and PI. Double negative cells (green) are considered vital, Annexin-Vpos (orange) are considered in apoptosis and double positive cells (red) are considered dead. Expression of CD11b and CD66b was analysed by FACS analysis after incubation of neutrophil granulocytes with TNF-α or medium only for 6 h (A), 24 h (B), 48 h (C) and 72 h (D). Every dot plot or histogram consists of 10.000 events. Gating cut-off was set by measurement of unstained controls for PI and Annexin-V or iso controls for CD11b and CD66b. [file Image_3.tif]

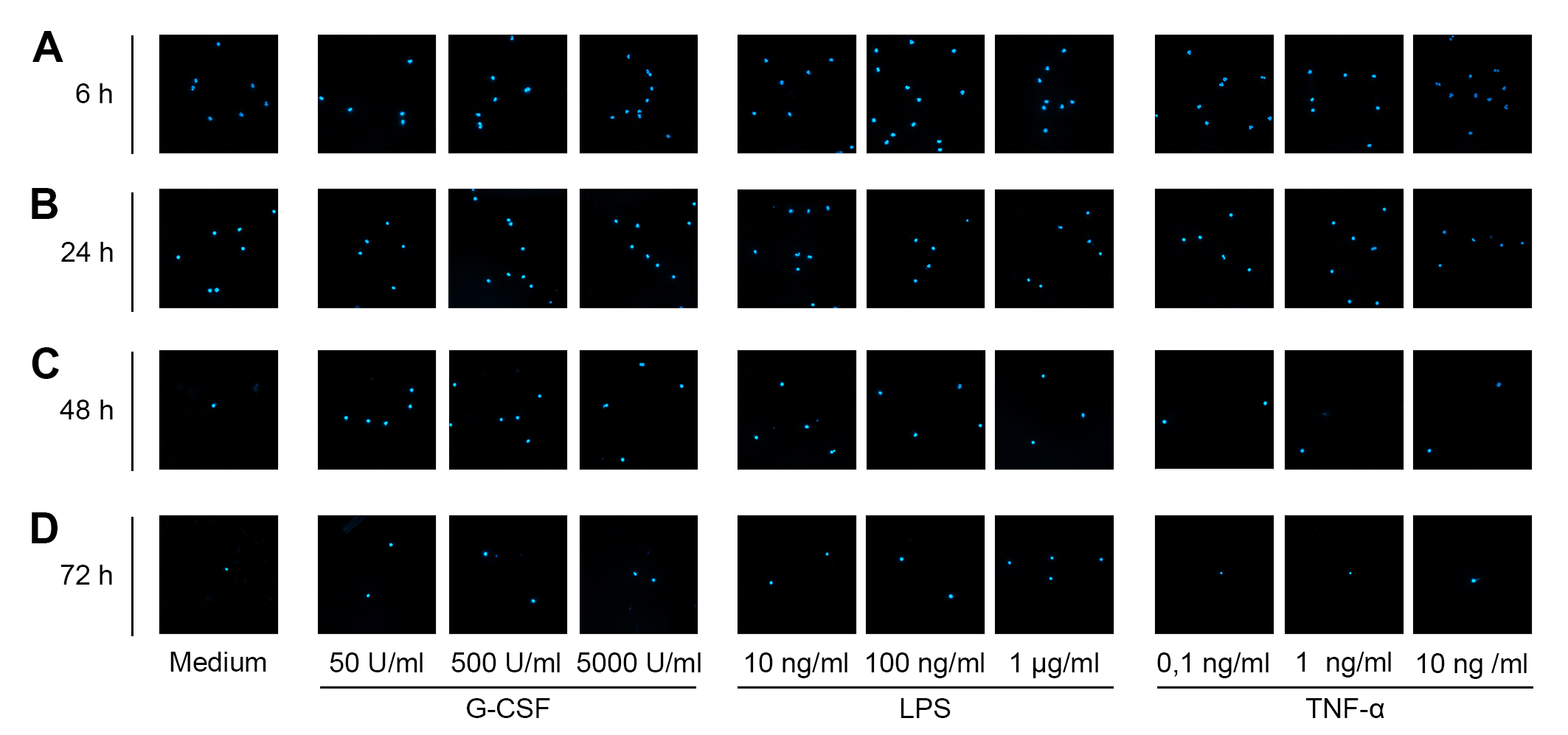

Supplement: Supplementary Figure 4 — Immunofluorescence imaging of neutrophils. Immunofluorescence imaging showed no extracellular DNA of neutrophils incubated with G-CSF, LPS or TNF-α. After fixation, DNA was stained with 1 µg/mL DAPI (blue). Images represent areas with comparable cell density at 40× magnification. [file Image_4.tif]
