# Supplementary material for: NET Release of Long-Term Surviving Neutrophils
Source: Front Immunol. 2022 Feb 15;13:815412. doi: 10.3389/fimmu.2022.815412 (PMC8887621; doi:10.3389/fimmu.2022.815412)
Supplement: Supplementary Table 1 — Cell count after incubation time is displayed in relation to 3,5 × 10^5^ cells seeded at the beginning. Neutrophils were incubated with mentioned doses of G-CSF, LPS, TNF-α or Medium only and counted after 6 h, 24 h, 48 h and 72 h of incubation with a haemocytometer. Values represent means ± SD of n = 3. [file Table_1.docx]

|  | | | | | | | | |
| --- | --- | --- | --- | --- | --- | --- | --- | --- |
| **Cells after incubation over 72 h** | | | | | | | | |
| Treatment | 6 h | | 24 h | | 48 h | | 72 h | |
|  | Mean | ± SD | Mean | ± SD | Mean | ± SD | Mean | ± SD |
| Medium | 100 % | ± 10 % | 88 % | ± 3 % | 86 % | ± 11 % | 82 % | ± 11 % |
| G-CSF 50 U/ml | 99 % | ± 9 % | 95 % | ± 14 % | 97 % | ± 5 % | 86 % | ± 10 % |
| G-CSF 500 U/ml | 100 % | ± 16 % | 100 % | ± 4 % | 100 % | ± 10 % | 100 % | ± 2 % |
| G-CSF 5000 U/ml | 100 % | ± 15 % | 100 % | ± 7 % | 99 % | ± 14 % | 99 % | ± 5 % |
| LPS 10 ng/ml | 93 % | ± 8 % | 94 % | ± 8 % | 80 % | ± 6 % | 65 % | ± 9 % |
| LPS 100 ng/ml | 90 % | ± 5 % | 88 % | ± 4 % | 76 % | ± 7 % | 70 % | ± 8 % |
| LPS 1 µg/ml | 91 % | ± 9 % | 76 % | ± 5 % | 85 % | ± 5 % | 67 % | ± 7 % |
| TNF-α 0,1 ng/ml | 100 % | ± 11 % | 100 % | ± 3 % | 80 % | ± 6 % | 89 % | ± 5 % |
| TNF-α 1 ng/ml | 99 % | ± 12 % | 94 % | ± 3 % | 91 % | ± 10 % | 81 % | ± 8 % |
| TNF-α 10 ng/ml | 90 % | ± 4 % | 77 % | ± 7 % | 76 % | ± 12 % | 84 % | ± 1 % |
|  |  |  |  |  |  |  |  |  |
